# Supplementary material for: The development of visual acuity and crowding reveals the slow fine-tuning of foveal vision
Source: Sci Rep. 2025 Dec 24;16:3234. doi: 10.1038/s41598-025-33169-2 (PMC12830654; doi:10.1038/s41598-025-33169-2)
Supplement: Supplementary file 1 — Supplementary Material 1 [file 41598_2025_33169_MOESM1_ESM.pdf]

Supplementary information for *The development of visual acuity and crowding reveals the slow fine-tuning of foveal vision*

John A. Greenwood, Marilia Kyprianou, & Tessa M. Dekker

The influence of attentional lapses

Given suggestions that frequent attentional lapses can hinder the detection of visual abilities[1], we examined the influence of these lapses on our data. We can quantify attentional lapses through the catch trials, whereby stimuli were presented at sizes three times larger than the QUEST estimate of threshold for that trial. Here we removed participants achieving less than 100% correct on these trials, independently for each of the unflanked and flanked-same conditions. There were not a great number of children who failed to meet this criteria in either case, though lapses were slightly higher in the flanked-same trials (as shown in Figure 3A). Amongst the 3-4 year olds, none were removed from the unflanked condition, with 2 removed from the flanked-same to leave 8. One 5-6 year old was removed from both conditions, leaving 21. One 7-8 year old was removed from the unflanked and two from the flanked-same, leaving 23 and 22, respectively. Of the 9-10 year olds, 3 were removed from the unflanked and 1 from the flanked-same, leaving 11 and 13, respectively. No 11-12 year olds were removed from the unflanked condition, and just one from the flanked-same to give 19 and 18, respectively. Six adults were removed from the unflanked condition to leave 24, and two from the flanked-same condition to give 28.

Thresholds from the remaining participants are shown in Figure S1. Mean values did not vary appreciably from those presented in the main analysis in Figure 3C. As before, isolated performance was significantly elevated relative to adult thresholds in the youngest children at 3-4 years (t_{32} = 4.853, $p < 0.0001$, d = 1.83), with no significant difference for any age groups beyond this (5-6 years: t_{43} = 1.495, p = 0.142, d = 0.45; 7-8 years: t_{45} = 1.575, p = 0.122, d = 0.46; 9-10 years: t_{33} = -0.872, p = 0.390, d = 0.32; 11-12 years: t_{41} = 1.239, p = 0.222, d = 0.38). The addition of flankers similarly produced clear elevations in threshold, which were significantly larger than adults at 3-4 years (t_{34} = 7.783, $p < 0.0001$, d = 3.12) and at 5-6 years (t_{47} = 3.141, p = 0.003, d = 0.91), but not at 7-8 years (t_{48} = 0.500, p= 0.619, d = 0.14) or beyond (9-10 years: t_{39} = 0.728, p = 0.471, d = 0.24; 11-12 years: t_{44} = 0.548, p= 0.586, d = 0.17). Given that the removal of participants with catch-trial performance below 100% does not alter the pattern of results, we conclude that differences

in attentional lapse rates with age are unlikely to have played a major role in the developmental trajectories for acuity and crowding.

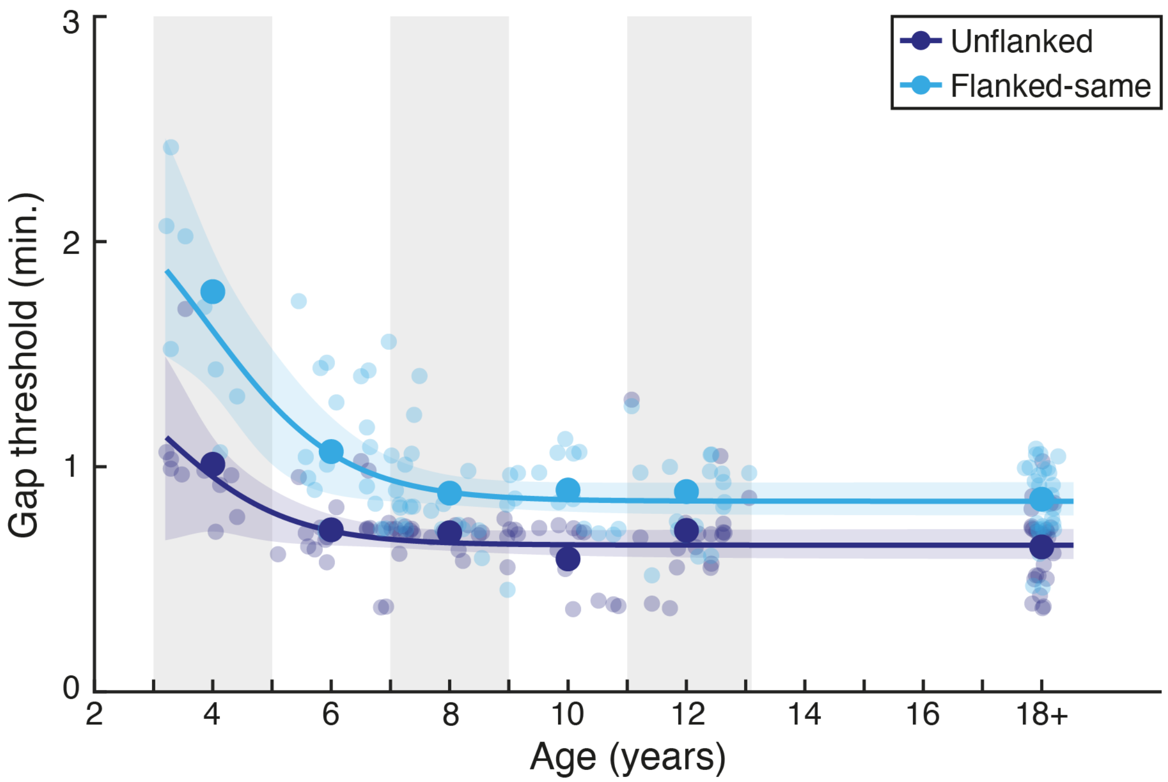

Figure S1. Gap-size thresholds plotted by age after filtering to remove individuals with performance on catch trials that was below 100% correct. As in Figure 3C, thresholds are plotted in minutes of arc, with individuals shown as small points and the mean in each age range (shown via grey/white bands) as large points, separately for the unflanked (dark blue) and flanked-same (light blue) conditions. Adult data has been collapsed to fall near 18 years. Lines show the best fitting logistic function to each dataset, with shaded regions showing the 95% range of fits to 1000 bootstrapped samples.

Comparisons to prior studies using scaling vs. spacing approaches

Prior reports of the age at which crowding reaches adult levels, summarised in Figure 1, show an apparent divergence between studies where elements were scaled (as in the current study) and those using fixed stimulus sizes where inter-element separation was varied directly using elements of a fixed size (typically set as a multiple of unflanked acuity, using values measured prior to the measurement of crowding). Because two of our prior studies have taken these distinct approaches, we compared the resulting estimates of crowding to consider the influence of these measurement approaches.

The direct manipulation of inter-element spacing was previously used by Greenwood *et al*[2] using the same VacMan stimuli to examine crowding in children aged 4-8. Using stimuli of

a fixed size (2.5 times above acuity thresholds), Greenwood *et al*[2] varied the centre-to-centre separation between elements to find the separation where performance reached 62.5% correct. Trial-by-trial data was extracted from this study and re-fit with psychometric functions for comparison to the current study. To consider the reliability of our estimates, we can further compare our values with a recent study[3], where a scaling method similar to the current study was used with children aged 3-8 years. Because Kalpadakis-Smith *et al*[3] used a higher threshold of 80% correct to measure the full extent of crowding, their trial-by-trial data was extracted and re-fit to obtain thresholds at 62.5% correct. Values from these two studies can then be compared with those of the current study by calculating the centre-to-centre separation of the elements in the current study at threshold. We can do so by multiplying our gap-size thresholds by 5 (to give stimulus diameter) and again by 1.1 (the scaled centre-to-centre separation) to give thresholds for the centre-to-centre separation between elements in minutes of arc.

The resulting individual values are plotted against those of the current study in Figure S2. Values from the current study again show a clear developmental trajectory, with thresholds from Greenwood *et al*[2] and Kalpadakis-Smith *et al*[3] largely interspersed amongst these values to follow this trajectory, aside from a small number of outliers. We conclude that thresholds obtained using a scaled approach (as in the current study and previously[3]) are comparable to those that vary inter-element separation directly[2]. If anything, the direct manipulation of inter-element separation would appear to produce more variable data, with two children from Greenwood *et al*[2] in particular showing very large critical spacing estimates for their age. We suspect that this approach could lead to problems if acuity thresholds were over-estimated, which would make it difficult for children to reach ceiling performance in the flanked conditions (since stimuli may be too small to reach a high level of recognition, even at the largest separations). As the scaling approach measures flanked and unflanked performance independently, there are no such issues with the cross-dependency of thresholds.

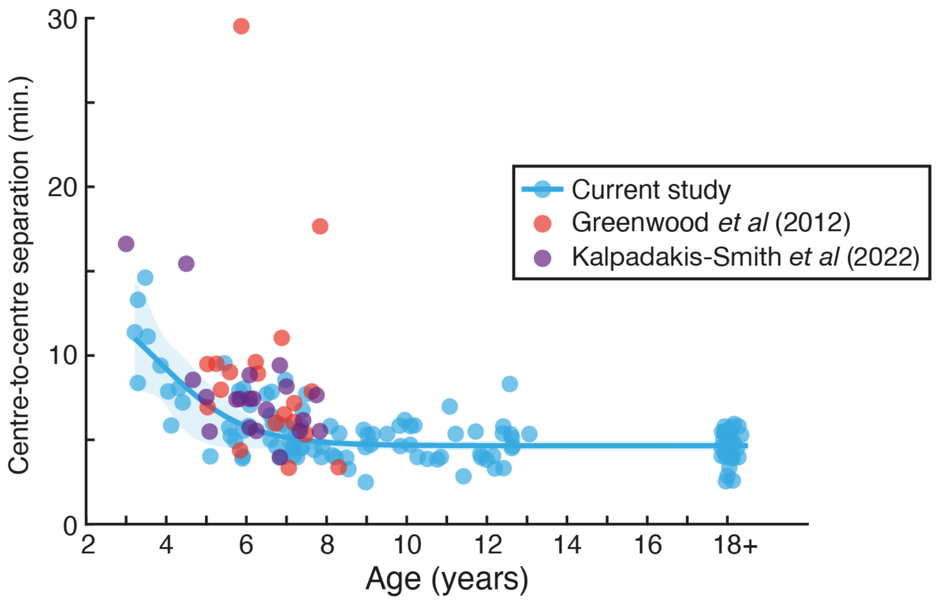

Figure S2. A comparison of different approaches to measure crowding. Thresholds from the flanked-same condition of the current study are shown in light blue, here expressed as the centre-to-centre separation between elements at threshold (in minutes of arc), along with the best-fitting logistic function for these values and a shaded region showing the 95% range of fits to 1000 bootstrapped samples. Thresholds are shown in equivalent centre-to-centre separation units from Greenwood *et al*[2] in red (which measured inter-element separation directly) and Kalpadakis-Smith *et al*[3] in purple (which used the same scaling approach as the current study). Each point represents an individual child.

Meta-analysis of the developmental trajectory of crowding

To examine the correspondence between our estimates of the developmental trajectory of crowding and those of prior studies, we sought to compare these estimates directly. As well as the studies shown in Figure 1 (which only includes published studies with direct statistical comparison to adult data), here we also included studies without statistical comparison to adult values and unpublished work, allowing a broader view of the literature. Values from prior studies were extracted from the figures of each publication using the GRABIT tool for MATLAB. Mean values were taken in all but one case where individual values were plotted (as below). For comparison to our dataset, values were plotted either at the reported age values or at the middle of range values, where used. For each comparison, individual values from the present study were converted to the same units of measurement and re-fit with a logistic function. To compare values against those of the current study, we derived error terms for the logistic fit to our dataset (separately after conversion to each measurement unit) by taking the 95% range of values obtained by fitting the logistic function to 1000 bootstrapped samples of the data.

A common metric used in prior studies is to report crowding as multiples of acuity thresholds, measuring the elevation in crowding relative to baseline acuity levels. This approach was first taken by Atkinson *et al*[4] using the Cambridge Crowding Cards (n=71 overall). Figure S3A shows that these values tend to be slightly lower than ours, consistent with the broader spacing of their elements (1.5× vs. 1.1× element size in the present study), which has been shown to underestimate the amount of crowding[5]. Thresholds nonetheless follow roughly the same developmental trend as the current study and fall within the 95% range of our fitted values for both sets of children. Similar measures were taken by Huurneman *et al*[6] using acuity charts (n=75). Their most-closely spaced condition (1.0× element size) is also plotted in Figure S3A, which again follows largely the same developmental trend. Together, these estimates of crowded elevation are not widely divergent from those of the present study. Of note however is the individual variability in these estimates – whereas the individual values plotted separately for unflanked and flanked-same conditions clearly follow the developmental trajectory in Figure 3, their greater dispersal here makes the trajectory harder to discern. We suggest that it is better to take independent measures of performance and to compare them across conditions than to use estimates that combine multiple sources of error.

We can also compare our thresholds to prior work measuring crowding as the log difference between flanked and unflanked thresholds. Our data can be converted to this format by converting gap-size thresholds in minutes of arc to log units and then subtracting flanked thresholds from unflanked. Values from Norgett and Siderov[7] obtained with a standard target-flanker arrangement (a letter target with 4 flankers, as in the current study, with n=89) are plotted in Figure S3B. These values lie close to ours, with the youngest children falling within our 95% range, though both older children and adults sit slightly below ours, likely driven by the wider spacing of 1.5× element size underestimating crowding levels, as above. Similar estimates can be seen in an earlier study using the same approach (n=103)[8], also plotted in Figure S3B. Also included in this subplot is data from Facchin *et al* (n=252)[9], who measured logMAR acuity using letter-chart arrangements at a range of inter-element spacings. For comparison, log thresholds were taken from the widest (2.0× element size) and narrowest (1.125×) centre-to-centre spacing conditions, which were then subtracted to give log difference values. These estimates again agree closely with our own.

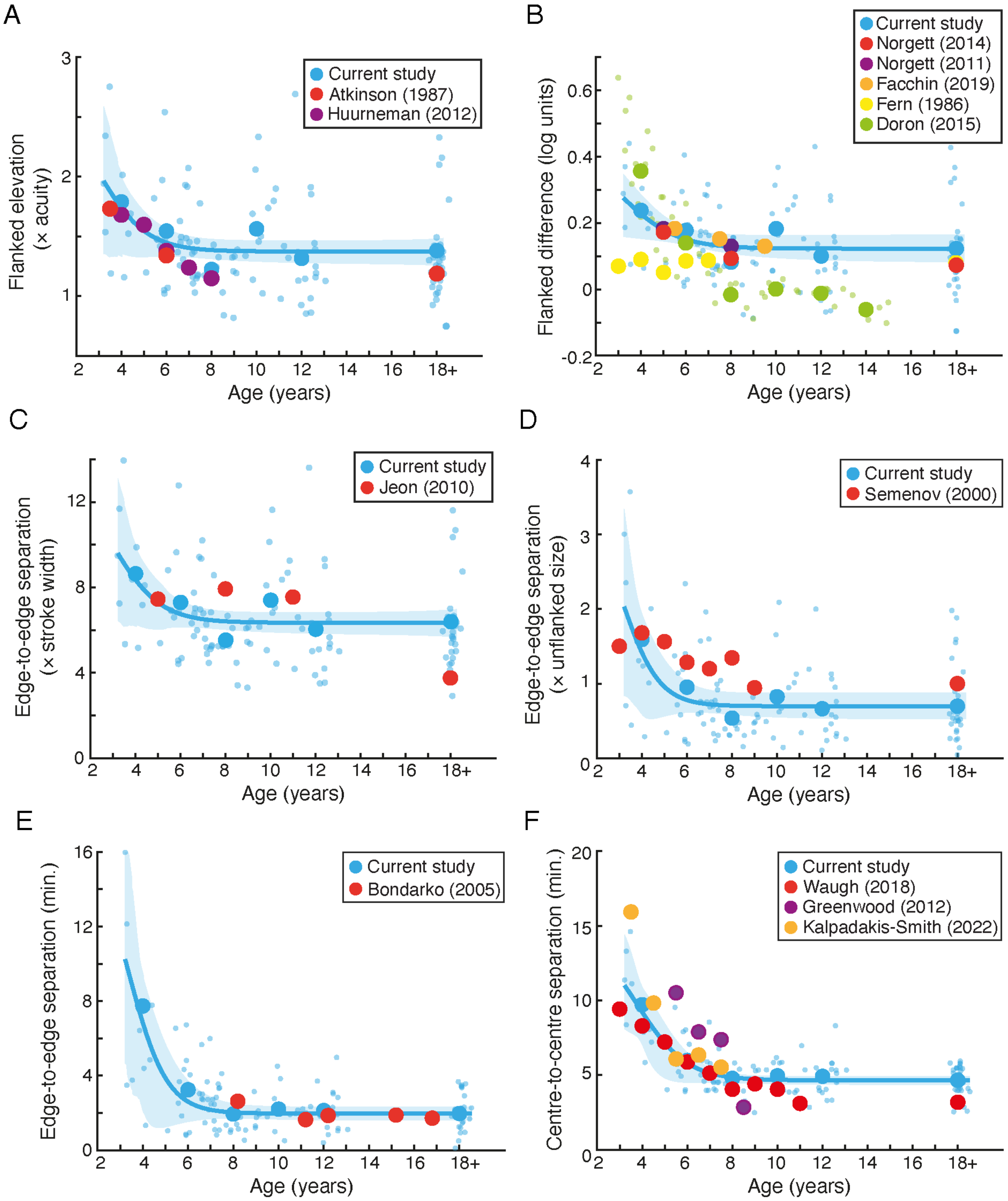

Figure S3. Comparison to prior datasets, plotted as a function of age on the x-axis. **A.** Crowding expressed in multiples of unflanked thresholds, with individuals shown as small blue dots and means for each age range (as in Figure 3) shown as large blue points. Comparison data is shown from Atkinson *et al*[4] in red and Huurneman *et al*[6] in purple. Lines show the best-fitting logistic function with shaded region indicating the 95% range of bootstrapped values. **B.** Crowding expressed as the difference between flanked and unflanked thresholds in log units, plotted against values from Norgett and Siderov[7], Norgett and Siderov[8], Facchin *et al*[9], Fern *et al*[10], and Doron *et al*[11]. **C.** Crowding expressed as the estimated edge-to-edge separation between elements, in multiples of stroke width, with comparison to data from Jeon *et al*[12] in red. **D.** Crowding as edge-to-edge separation in multiples of element size, plotted against data from Semenov *et al*[13]. **E.** Crowding as edge-to-edge separation in minutes of arc, plotted against data from Bondarko and Semenov[14]. **F.** Crowding as the centre-to-centre separation between elements in minutes of arc, plotted against data from Waugh *et al*[15], Greenwood *et al*[2], and Kalpadakis-Smith *et al*[3].

Differences emerge in the log difference values reported by Fern et al[10], who used a C vs. O target discrimination task with letters either in isolation or flanked by four widely spaced flanker bars (at a centre-to-centre separation of approximately 1.5× target diameter, with n=121; Figure S3B). Although on its own their flanked data shows a clear developmental decline, their log difference values (with acuity subtracted) are uniformly low and largely unchanged with age. We suspect that this reflects an underestimation of crowding levels, for two reasons. First, the use of bars as flanker stimuli have been shown to underestimate crowding[5,7], likely through the decreased target-flanker similarity of the bars to the target letters[16]. The large inter-element spacing would further decrease the level of crowding measured with these elements[5]. Differences are also evident in the values obtained by Doron *et al*[11], measured with letter-chart arrays of Tumbling-E stimuli (n=46). Because individual values were plotted in this study, we recorded these and calculated mean crowding levels within each of the age ranges used in the current study, as plotted in Figure S3B. Although this approach gives an estimate for the age of maturity that is very similar to that of the present study, the estimates of crowding are higher than ours for the youngest children, likely due to the use of high numbers of elements causing uncertainty in the youngest children. An undershoot in crowding levels is also visible in the oldest children, likely driven by the letter-chart arrangement whereby each element is flanked by a maximum of 2 elements (depending on the position of the letter in each line). Although the absolute values diverge from ours in this instance, the developmental trajectory nonetheless remains largely the same.

The study with the oldest age of maturity for crowding comes from Jeon *et al*[12], who took measures of the edge-to-edge separation between target and flanker elements, with elements presented at a size 1.2× acuity (measured initially) and varied in their separation (n=78). We can convert our values for comparison to theirs with some assumptions. The scaling of our elements meant that the edge-to-edge separation was always 0.1× the element size. We can however estimate what our values might have been had we varied spacing using fixed-size elements by taking the centre-to-centre separation between elements at threshold and subtracting the diameter of elements at the acuity threshold multiplied by 1.2, before dividing by the gap-size threshold to give units of stroke width. The resultant values are plotted against those of Jeon *et al*[12] in Figure S3C. Only 1 of 3 estimates of childhood crowding coincide with our 95% range, though the largest discrepancy occurs in the adult data, where the estimates from Jeon *et al*[12] are considerably lower than those of

the adults measured with our approach. One possibility is that the adult sample collected by Jeon *et al*[12] were more experienced with psychological testing than ours. Differences in the nature of the testing procedures used in children and adults could also give rise to this divergence. In comparison, our sample included predominantly adults who were inexperienced with psychological testing, with the same testing conditions used for both adults and children, and independent measures of the two abilities. The relative nature of these estimates (with flanked thresholds divided by unflanked) also makes it difficult to assess whether the difference reflects changes in crowding, acuity, or both.

Similar calculations allow comparison with the data of Semenov *et al*[13], who varied the edge-to-edge separation between target and flanker bar elements presented at a size equal to acuity thresholds (n=141). Values were reported as edge-to-edge separation in multiples of unflanked whole-element size, which we can again calculate for our thresholds by taking the centre-to-centre separation between elements at threshold and subtracting the diameter of elements at the acuity threshold, before dividing by element diameter to give multiples of unflanked size. Semenov *et al*[13] used a subjectively determined high level of performance to take threshold, which we attempted to match by taking thresholds at 90% correct (instead of 62.5% correct elsewhere). As shown in Figure S3D, the estimates of Semenov *et al*[13] are elevated relative to ours in both children and adults, and show a period of continued elevation. Given the derivation of thresholds at high performance levels however, it is possible that some of this prolongation is due to attentional lapses, which are a primary cause of disrupted performance at the upper end of the psychometric function in particular[1,17], which would also make the subjective estimation of asymptotic performance difficult.

Bondarko and Semenov[14] conducted similar procedures to Semenov *et al*[13], measuring the edge-to-edge separation between target and flanker elements, with elements presented at a size equal to acuity thresholds and varied in their separation (n=292). Here, values were reported as edge-to-edge separation in minutes of arc, which are more easily compared to ours (with the above assumptions regarding stimulus size), as plotted in Figure S3E. Here although their youngest 8-year-old participants show a slight elevation relative to ours, the bulk of the measurements align well with that of the current dataset. The age-range in our data nonetheless reveals that far greater changes in the developmental trajectory are evident before the age of 8 years. As with the estimates of Semenov *et al*[13], the use of

subjective fitting procedures to asymptotic data ranges may have led to the slight elevation in performance seen here with the 8 year old participants. Estimating a lower performance criterion may alter these estimates.

Finally, unpublished data from Waugh *et al*[15] allows comparison to our dataset with fewer assumptions. Waugh *et al* used narrow number stimuli that were scaled using QUEST to obtain size thresholds. These estimates were converted to critical spacing values (n=241), similar to our computations shown in Figure S2. Taking their 3-element configuration (with one target and two flankers) as the closest to the majority of studies above, these estimates of the centre-to-centre separation between target and flanker elements at threshold can be compared to ours (Figure S3F). Both show a highly similar rate of decline in the size of these interference zones with age. We can further compare the data from Greenwood et al[2] (n=19) and Kalpadakis-Smith et al[3] (n=20) by binning these values into comparable age ranges and taking the means within each bin, also shown in Figure S3F. Values from Kalpadakis-Smith et al[3] are again highly similar to both the current study and estimates from Waugh *et al*[15], while those from Greenwood et al[2] follow the same trend, though with increased variability, as noted above. The overall consistency of these developmental trajectories is nonetheless evident across these studies using similar approaches.

To combine these disparate metrics of crowding for meta-analysis, we used our dataset in each case as a reference. Each of the above 13 datasets were thus normalised by subtracting the minimum value from the mean values across age in our dataset (after conversion into the relevant metric), before dividing by the maximum mean value of our dataset. This set our dataset to span the range 0-1, with other datasets varying around this. The resultant values could then be combined in the same space. Mean values were taken within similar age ranges to those of the current study, which gave 19 estimates for 3-4 year-olds, 16 for 5-6 year-olds, 15 for 7-8 year-olds, 6 for 9-10 year-olds, 4 for 11-12 year-olds, 3 for 13-17 year-olds and 7 adult comparisons. Data for this meta-analysis are plotted in Figure 4 of the main text.

The analyses presented in Figure 4 of the main text include multiple contributions from individual studies in some of the age ranges. We also examined the pattern of data after this non-independence of individual datapoints was removed by averaging datapoints from individual studies within each of the above age ranges. This analysis restricted the

contribution of each study to a maximum of 1 point per age group. Results from this analysis are plotted in Figure S4, which show a very similar developmental trend to that in Figure 4 of the main text. Crowding levels were again highest in the 3-4 year age range, which differed significantly from adult levels (t_{16}= 4.423, $p < 0.0001$, d = 2.14). These levels remained significantly elevated at 5-6 years with a large effect size (t_{15} = 3.141, p= 0.007, d = 1.55), but dropped to levels that did not differ significantly from adults at 7-8 years (t_{17} = 1.339, p= 0.198, d = 0.64), and remained equivalent at 9-10 years (t_{10} = 0.676, p= 0.514, d = 0.40), 11-12 years (t_8 = -0.601, p= 0.564, d = 0.41) and 13-17 years (t_6 = -1.389, p= 0.207, d = 1.11). We conclude that the maturation of crowding at 7-8 years is a reliable feature of this meta-analytic dataset.

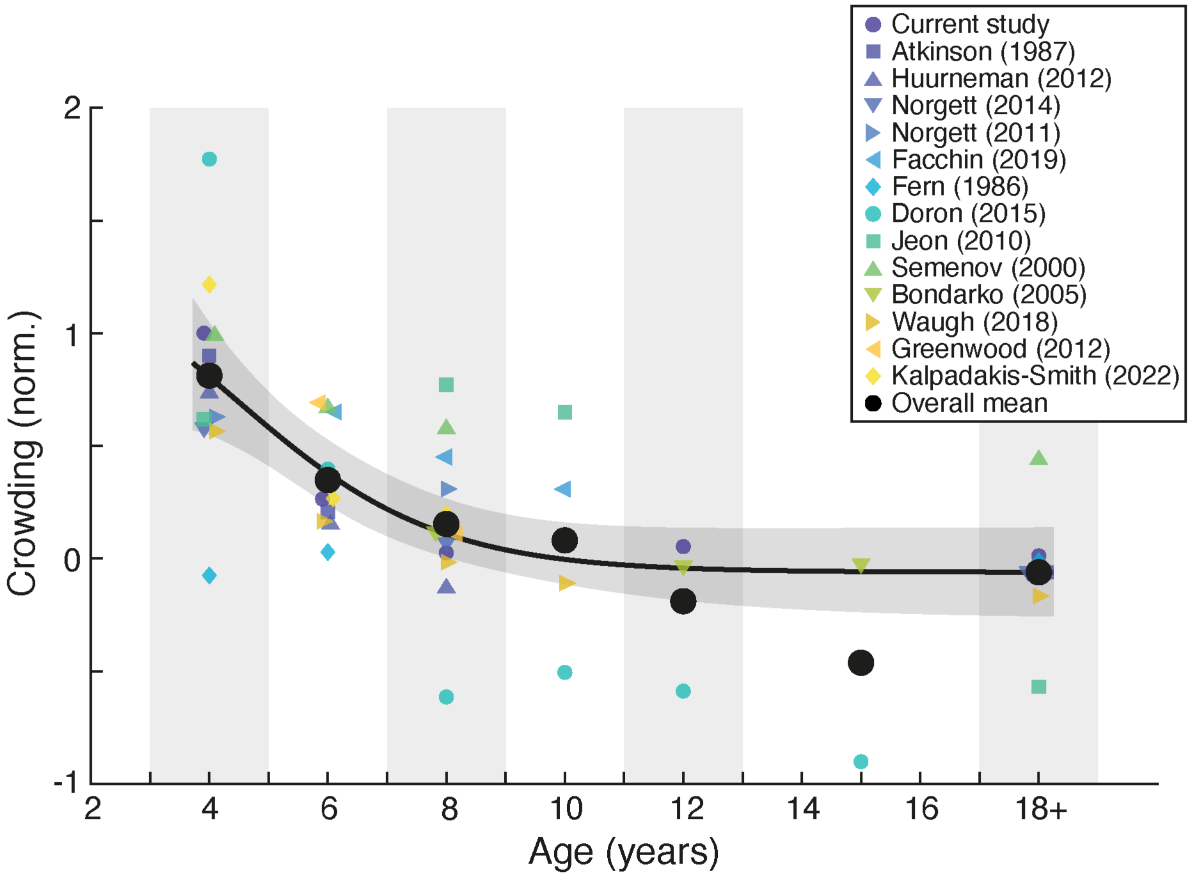

Figure S4. A meta-analysis of studies measuring the developmental trajectory of crowding. As in Figure 4 of the main text, crowding is plotted in normalised units, as a function of age on the x-axis. Small symbols show estimates from individual studies (here averaged within each age range prior to plotting), while the large black points show the mean within each age range (indicated via shaded regions). The black line is the best-fitting logistic function fit to all studies, with shaded region indicating the 95% range of the fits to 1000 bootstrapped samples. Some thresholds have been displaced on the x-axis for visibility.

References

1 Dekker, T. M., Farahbakhsh, M., Atkinson, J., Braddick, O. J. & Jones, P. R. Development of the spatial contrast sensitivity function (CSF) during childhood: Analysis of previous findings and new psychophysical data. *Journal of Vision* **20**, 4-4 (2020).

2 Greenwood, J. A. *et al*. Visual acuity, crowding and stereo-vision are linked in children with and without amblyopia. *Investigative Ophthalmology & Visual Science* **53**, 7655-7665 (2012).

3 Kalpadakis-Smith, A. V., Tailor, V. K., Dahlmann-Noor, A. H. & Greenwood, J. A. Crowding changes appearance systematically in peripheral, amblyopic, and developing vision. *Journal of Vision* **22(6):3**, 1-32 (2022).

4 Atkinson, J., Anker, S., Evans, C. & McIntyre, A. in *Transactions of the Sixth International Orthoptic Congress* (ed M. Lenk--Schaefer) 482-486 (British Orthoptic Society, 1987).

5 Song, S., Levi, D. M. & Pelli, D. G. A double dissociation of the acuity and crowding limits to letter identification, and the promise of improved visual screening. *Journal of Vision* **14**, 1-37 (2014).

6 Huurneman, B., Boonstra, F. N., Cillessen, A. H. N., van Rens, G. & Cox, R. F. A. Crowding in central vision in normally sighted and visually impared children aged 4 to 8 years: the influence of age and test design. *Strabismus* **20**, 55-62 (2012).

7 Norgett, Y. & Siderov, J. Foveal crowding differs in children and adults. *Journal of Vision* **14**, 23-23 (2014).

8 Norgett, Y. & Siderov, J. Crowding in children's visual acuity tests—Effect of test design and age. *Optometry and Vision Science* **88**, 920-927 (2011).

9 Facchin, A., Maffioletti, S., Martelli, M. & Daini, R. Different trajectories in the development of visual acuity with different levels of crowding: The Milan Eye Chart (MEC). *Vision Res* **156**, 10-16 (2019).

10 Fern, K. D., Manny, R. E., Davis, J. R. & Gibson, R. R. Contour interaction in the preschool child. *Optometry and Vision Science* **63**, 313-318 (1986).

11 Doron, R., Spierer, A. & Polat, U. How crowding, masking, and contour interactions are related: A developmental approach. *Journal of Vision* **15**, 5-5 (2015).

12 Jeon, S. T., Hamid, J., Maurer, D. & Lewis, T. L. Developmental changes during childhood in single-letter acuity and its crowding by surrounding contours. *J Exp Child Psy* **107**, 423-437 (2010).

13 Semenov, L. A., Chernova, N. D. & Bondarko, V. M. Measurement of visual acuity and crowding effect in 3–9-year-old children. *Human Physiology* **26**, 16-20 (2000).

14 Bondarko, V. M. & Semenov, L. A. Visual acuity and the crowding effect in 8-to 17-year-old schoolchildren. *Human Physiology* **31**, 532-538 (2005).

15 Waugh, S. J., Pelli, D. G., Álvaro, L. & Formankiewicz, M. A. Crowding distance in healthy children [version 1; not peer reviewed]. *F1000Research* **7**, 707 (poster) (2018).

16 Toet, A. & Levi, D. M. The two-dimensional shape of spatial interaction zones in the parafovea. *Vision Res* **32**, 1349-1357 (1992).

17 Manning, C., Jones, P. R., Dekker, T. M. & Pellicano, E. Psychophysics with children: Investigating the effects of attentional lapses on threshold estimates. *Attention, Perception, & Psychophysics*, 1-14 (2018).
